# MicroRNA 196B regulates FAS-mediated apoptosis in colorectal cancer cells

**DOI:** 10.18632/oncotarget.3066

**Published:** 2014-12-26

**Authors:** Ji-Su Mo, Khondoker Jahengir Alam, In-Hong Kang, Won Cheol Park, Geom-Seog Seo, Suck-Chei Choi, Hun-Soo Kim, Hyung-Bae Moon, Ki-Jung Yun, Soo-Cheon Chae

**Affiliations:** ^1^ Department of Pathology, School of Medicine, Wonkwang University, Iksan, Chonbuk, Republic of Korea; ^2^ Department of Surgery, School of Medicine, Wonkwang University, Iksan, Chonbuk, Republic of Korea; ^3^ Digestive Disease Research Institute, Wonkwang University, Iksan, Chonbuk, Republic of Korea

**Keywords:** Fas, MIR196B, Colorectal cancer, Apoptosis

## Abstract

Using miRNA microarray analysis, we identified 31 miRNAs that were significantly up-regulated or down-regulated in colon cancer tissues. We chose MIR196B, which was specifically up-regulated in colon cancer, for further study. We identified 18 putative MIR196B target genes by comparing between the mRNAs down-regulated in MIR196B-overexpressed cells and the assumed MIR196B target genes predicted by public bioinformatics tools. The association between MIR196B and *FAS* was verified in this study. FAS expression was constitutively elevated in normal human colorectal tissues. However, its expression was often reduced in human colorectal cancer. The decrease in FAS expression could be responsible for the reduction of apoptosis in colorectal cancer cells. In colorectal cancer tissue, we showed that MIR196B up-regulation was mutually followed by down regulation of FAS expression. We also showed that MIR196B directly repressed FAS expression in colorectal cells. Furthermore, anti-MIR196B up-regulated FAS expression and increased apoptosis in colorectal cancer cell lines. Our results suggest that the up-regulation of MIR196B modulates apoptosis in colorectal cancer cells by partially repressing FAS expression and that anti-MIR196B could be a potential candidate as an anti-cancer drug in colorectal cancer therapy.

## INTRODUCTION

Colorectal cancer (CRC) is one of the most common cancers worldwide [1], and the third leading cause of cancer-related mortality in the United States [2]. Its etiology is very complex and not fully understood. Numerous epidemiological and biological studies have suggested that various risk factors, including nutrition, physical inactivity, obesity, and diabetes, play a critical role in the etiology of CRC [3, 4]. Most sporadic colorectal cancers are associated with inflammation and inflammatory diseases, such as inflammatory bowel disease (IBD) [5]. Several genetic factors, including microRNAs (miRNAs), are also thought to contribute to colorectal cancer risk. Much evidence suggests that miRNAs are important regulators of oncogenesis [6, 7].

miRNAs are endogenously synthesized, short, noncoding RNA molecules of approximately 19-24 nucleotides. miRNAs contribute to the post-transcriptional regulation of gene expression in multicellular organisms by controlling the stability and translation of target mRNAs [8]. miRNAs contribute to the regulation of crucial biological processes, such as cell proliferation, apoptosis, differentiation, and angiogenesis [9]. They are also implicated in the pathogenesis of various diseases as tumor suppressor genes or oncogenes [10, 11]. Therefore, characterization of miRNA expression patterns in cancer cells may have substantial value for disease diagnosis, prognosis, and therapy [12-14].

The members of the MIR196 gene family (MIR196A1, MIR196A2, and MIR196B) are transcribed from three different genes, which are located in homeobox (HOX) gene cluster regions in humans [15, 16]. The *MIR196A1* gene is located on Chr. 17q21.32 between the *HOXB9* and *HOXB10* genes. The *MIR196A2* gene is located between *HOXC10* and *HOXC9* on Chr. 12q13.13. The gene for *MIR196B* is located in an evolutionarily conserved region between *HOXA9* and *HOXA10* on Chr. 7p15.2. The mature nucleotide sequences of MIR196A1 and MIR196A2 are identical, whereas mature MIR196B differs from MIR196A by one nucleotide [16]. Previous studies suggests that MIR196 may play critical roles in normal development and cancer pathogenesis by targeting specific genes [17].

In this study, we measured miRNA expression in colon cancer tissues and normal colon tissues by miRNA microarray analysis. We detected 31 microRNAs that were specifically up-regulated or down-regulated in colorectal cancer tissues. Of them, MIR196B was chosen for detailed analysis and further study. mRNA microarray expression profiles of MIR196B-overexpressing colorectal cancer cell lines were generated to identify MIR196B target molecules. The list of MIR196B target genes was narrowed down by comparison with a database of candidate target genes predicted by bioinformatics programs. We identified FAS cell surface death receptor (*FAS*, also called *Apo1* or *CD95*) as a MIR196B target gene in colorectal cancer and verified their association between MIR196B and FAS in colorectal cancer cells.

## RESULTS

### miRNA expression profiling in colon cancer tissue

**Figure 1:**
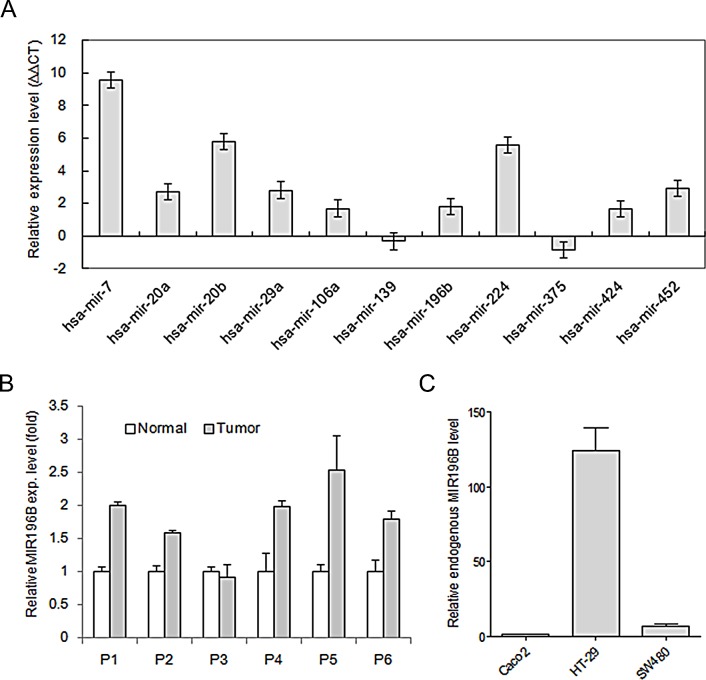
qRT-PCR analysis of miRNA expression in tissues and cells (A) The expression of 11 miRNAs was validated using four colon cancer tissue samples and matched normal colon tissue samples. miRNAs levels were normalized with colon specific RNU48. Values are presented as the relative levels (∆∆CT) of the miRNAs in colon cancer tissues. *P* < 0.01 for all miRNAs by paired t-test. (B) MIR196B expression in 6 pairs of colorectal cancer tissue samples and adjacent normal colorectal tissue samples. Values are presented as the fold-change in tumor tissue relative to normal tissue. P1, P2, P5, and P6 indicate patients with colon cancer. P3 and P4 indicate patients with rectal cancer. *P* < 0.05 for MIR196B by paired t-test. (C) The relative endogenous MIR196B expression levels in three colorectal cancer cell lines. Values are presented as the fold-change in HT29 or SW480 cells relative to Caco2 cells. *P* < 0.01 for MIR196B by t-test. Data represent the mean ± S.D. of three independent experiments, each carried out in duplicate.

We used miRNA microarray analysis to compare the expression of miRNA precursors in colon cancer tissue and normal colon tissue. Total RNA for miRNA chip analysis was obtained from four different patients with colon cancer. We initially compared miRNA expression in normal colon tissues and the matched colon cancer tissues in two independent experiments. We identified 31 miRNAs that were up-regulated or down-regulated more than two-fold in both independent experiments (Table 1). The results from the miRNA chip analysis were validated by qRT-PCR for 11 miRNAs (Fig. 1A). We selected one, MIR196B, which was up-regulated in human colorectal cancer tissue, for further investigation in this study (Fig. 1B).

**Table 1:** miRNAs differentially expressed in normal tissues and colon cancer tissues, as determined by miRNA microarray analysis

miRNA	Chromosomal location	Fold change
has-miR-7	9q21.32	6.58
hsa-miR-9-2	5q14.3	4.68
has-miR-15a	13q14.2	2.91
has-miR-17	13q31.3	6.87
has-miR-19a	13q31.3	4.17
has-miR-19b	13q31.3	4.14
has-miR-20a	13q31.3	7.05
has-miR-20b	Xq26.2	6.50
has-miR-21	17q23.1	3.60
has-miR-25	7q22.1	2.74
has-miR-27a	19p13.13	2.78
has-miR-29a	7q32.3	3.21
has-miR-29b	7q32.3	5.49
has-miR-34a	1p36.22	3.51
has-miR-34b	11q23.1	2.34
hsa-miR-93	7q22.1	3.14
has-miR-106a	Xq26.2	5.55
has-miR-106b	7q22.1	3.49
has-miR-146a	5p34	3.54
has-miR-139	11q13.4	0.53
has-miR-181c	19p13.13	2.99
has-miR-196b	7p15.2	7.18
has-miR-224	Xq28	11.34
has-miR-374	Xq13.2	2.88
hsa-miR-375	2q35	0.33
has-miR-424	Xq26.3	10.61
has-miR-452	Xq28	3.42
has-miR-590	7q11.23	3.49
has-miR-622	13q31.3	2.56
has-miR-630	15q24.1	2.94
has-miR-765	1q23.1	2.49

Four colon cancer tissue samples and matched normal colon tissue samples were analyzed twice with miRNA microarray. All differentially expressed miRNA had a *q* value < 0.01 (false-positive rate). All, except hsa-miR-139 and has-miR-375, were up-regulated in colon cancer tissues.

### mRNA expression profiling in MIR196B-overexpressing cells

To determine the levels of endogenous MIR196B in SW480, Caco2, and HT29 cells, we carried out qRT-PCR analysis using the total RNA isolated from the three different cell lines. As shown in Fig. 1C, the MIR196B level was lowest in Caco2 cells and highest in HT29 cells (Fig. 1C). To identify genes down-regulated by MIR196B overexpression, pre-MIR196B was transfected into SW480 and Caco2 cells. Increased expression of the miRNA 24 h after transfection in SW480 and Caco2 cells confirmed the transfection efficiency (Fig. S1A). Cells were harvested 48 h after transfection for mRNA expression profiling analysis with the Illumina HumanHT-12 v4 Expression BeadChip. We identified 67 genes whose expression was down-regulated 1.5-fold in cells overexpressing MIR196B (Table S1).

### MIR196B target gene identification

To identify MIR196B target genes, we compared the 67 candidate target genes identified by mRNA microarray analysis with candidate MIR196B target genes predicted by the TargetScan and miRWalk algorithms. Of the 67 genes, 18 were identified as putative targets of MIR196B (Table 2). Among them, we focused on *FAS* gene. To determine whether the expression levels of FAS in the colon cancer cell lines are comparable to the levels of MIR-196B, we carried out qRT-PCR or western blot analysis using the total RNAs or proteins isolated from SW480 and HT29 cells. HT29 cells show low *FAS* mRNA (Fig. S1B) and FAS (Fig. S1C) expression level compare to SW480 cells as MIR196B expression relatively higher in HT29 than SW480 cells. So, FAS expression might be depend on endogenous MIR196B expression in colon cancer cell lines.

**Table 2:** MIR196B target genes

Gene symbol	Accession	Gene name	Chromosome location	Functions
HOXB7	NM_004502.3	homeobox B7	17q21.3	transcription factor
HOXC8	NM_022658.3	homeobox C8	12q13.3	transcription factor
HOXA9	NM_152739.3	homeobox A9	7p15.2	transcription factor
GLTP	NM_016433.3	glycolipid transfer protein	12q24.11	transfer/carrier protein
LAMTOR5	NM_006402.2	late endosomal adaptor, MAPK and MTOR activator 5	1p13.3	regulate HBx activity
ANXA1	NM_000700.1	annexin A1	9q21.13	transfer/carrier protein
APEX2	NM_014481.2	APEX nuclease 2	Xp11.21	nucleic acid binding
FAS	NM_152877.1	cell surface death receptor	10q24.1	signal transduction
HAND1	NM_004821.1	heart and neural crest derivatives expressed 1	5q33	transcription factor
HOXA5	NM_019102.2	homeobox A5	7p15.2	transcription factor
HOXB6	NM_018952.4	homeobox B6	17q21.3	transcription factor
KCNIP3	NM_013434.4	Kv channel interacting protein 3, calsenilin	2q21.1	calcium binding protein
LRRC49	NM_017691.2	leucine rich repeat containing 49	15q23	-
MED10	NM_032286.2	mediator complex subunit 10	5p15.31	transcription factor
MRPL39	NM_017446.3	mitochondrial ribosomal protein L39	21q21.3	synthase and synthetase
SPRED1	NM_152594.1	sprouty-related, EVH1 domain containing 1	15q14	receptor
TGFB1I1	NM_015927.3	transforming growth factor beta 1 induced transcript 1	16p11.2	transcription cofactor
VSNL1	NM_003385.4	visinin-like 1	2p24.3	calcium binding protein

The target genes were identified by comparing mRNAs down-regulated in MIR196B-overexpressing cells with target genes predicted by public bioinformatics tools (TargetScan (http://www.targetscan.org) and miRWalk (http://www.umm.uni-heidelberg.de/apps/zmf/mirwalk/index.html).

### FAS is a target of MIR196B

**Figure 2:**
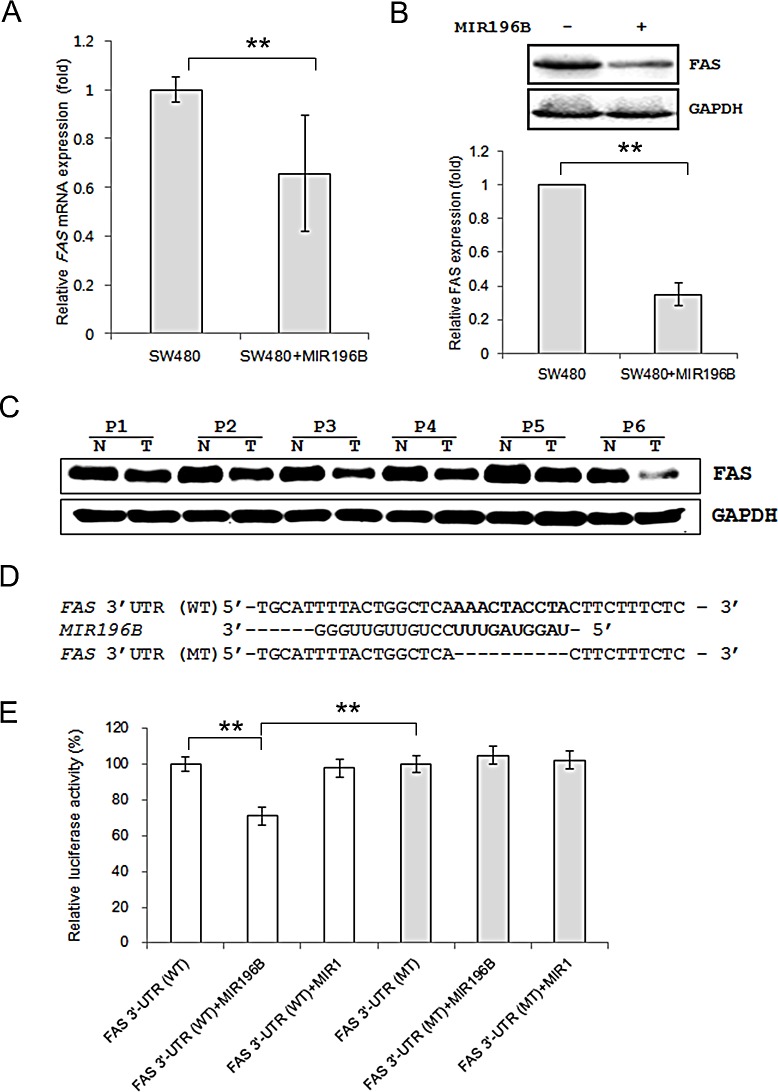
FAS is a direct target gene of MIR196B (A) qRT-PCR analysis of FAS expression in SW480 cells. SW480 cells were mock-transfected or transfected with pre-MIR196B. Cells were harvested 48 h after transfection. Total RNA was extracted and used for qRT-PCR. Values are presented as the fold-change in MIR196B-overexpressing cells relative to non-transfected cells. The experiment was performed in duplicate and repeated five times. (B) FAS protein levels in MIR196B-overexpressing cells and non-transfected cells. Protein was extracted 72 h after transfection for western blot analysis. (C) FAS expression in 6 pairs of colon cancer tissue samples and adjacent normal colon tissue samples. All samples are from patients with colon cancer. (D) Sequence alignment of the wild-type (WT) and mutated (MT) MIR196B target site in the 3′-UTR of *FAS*. A human *FAS* 3′ UTR containing the wild-type and mutant MIR196B binding sequence was cloned downstream of the luciferase reporter gene. (E) A luciferase reporter plasmid containing the WT or MT *FAS* 3′ UTR was co-transfected into SW480 cells with pre-MIR1 as a negative control or pre-MIR196B. Luciferase activity was determined using the dual luciferase assay. Results are shown as the relative firefly luciferase activity normalized to *Renilla* luciferase activity. Data assessed from three independent experiments and the *P* values were calculated by *t*-test (* *P* < 0.05; ** *P* < 0.01).

We investigated whether MIR196B regulated *FAS* mRNA and protein levels in SW480 cells. The *FAS* mRNA level was lower (0.66 fold) in SW480 cells transfected with pre-MIR196B than in un-transfected control cells (Fig. 2A). FAS protein expression was also down-regulated (0.35 fold) in MIR196B-overexpressing cells (Fig. 2B).

To demonstrate a direct interaction between the *FAS* 3′ UTR region and MIR196B, we cloned the WT *FAS* 3′ UTR region predicted to interact with MIR196B into a luciferase vector (Fig. 2D. Luciferase activity decreased by ~30% when cells were co-transfected with pre-MIR196B (Fig. 2E). As a control experiment, we cloned a mutated *FAS* 3′ UTR sequence lacking ten of the complementary bases. As expected, repression of luciferase activity was abolished when the interaction between MIR196B and its target 3′ UTR was disrupted (Fig. 2E). As additional control experiments, MIR1 instead of MIR196B was co-transfected with the WT and MT *FAS* 3′ UTR constructs. Transfection of pre-MIR1 did not affect the luciferase activity of either construct (Fig. 2E). These results suggest that MIR196B directly regulates *FAS* expression in colorectal cancer cells.

### FAS gene expression in human colorectal cancer

Given the findings described above, we investigated FAS expression in human colon cancer tissues and normal colon tissues by western blot analysis. FAS expression was down-regulated in all colon cancer tissues when compared to expression in normal colon tissues (Fig. 2C).

### MIR196B down-regulates FAS-mediated caspases in SW480 cells

**Figure 3:**
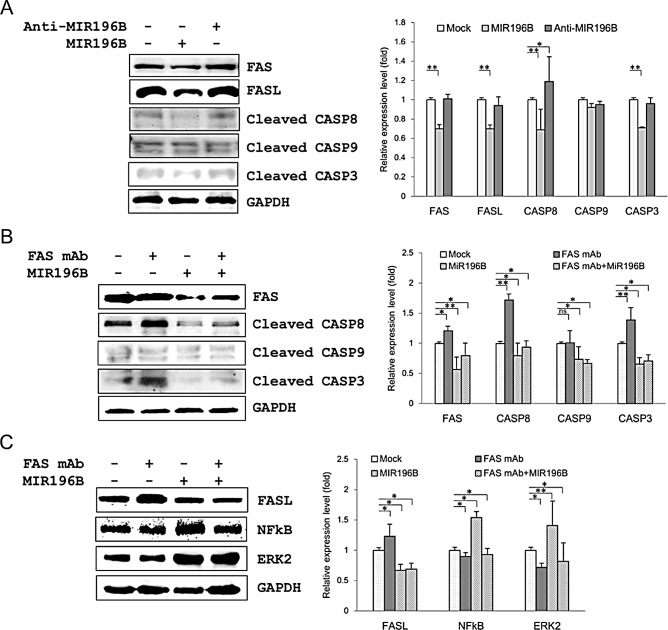
MIR196B regulates FAS-mediated apoptosis in SW480 cells (A) Western blot analyses of FAS, FasL, and FAS-mediated molecules in MIR196B- or anti-MIR196B-transfected SW480 cells. Proteins were extracted 72 h after transfection for western blot analysis. The data shown were normalized to GAPDH levels and are presented as fold-change in MIR196B- or anti-MIR196B-transfected cells relative to mock-transfected cells. The experiment was repeated four times. (B) Western blot analyses of FAS and FAS-mediated molecules in FAS mAb-treated or MIR196B-transfected SW480 cells. (C) Western blot analyses of FasL, NF-κB, and ERK2 in FAS mAb-treated or MIR196B-transfected SW480 cells. Protein was extracted 72 h after MIR196B transfection or FAS mAb treatment or 48 h after MIR196B transfection into cells cultured with FAS mAb for 24 h. The data shown were normalized to GAPDH levels and are presented as the fold-change in MIR196B-transfected or FAS mAb-treated cells relative to mock-treated cells. Data assessed from four independent experiments and the *P* values were calculated by *t*-test (* *P* < 0.05; ** *P* < 0.01; ns = not significant).

To further define the functional interaction between MIR196B and FAS in SW480 cells, we investigated the expression of proteins involved in the FAS-mediated apoptotic pathway, such as active (cleaved) caspase 8 (CASP8) active (cleaved) caspase 3 (CASP3), as well as the expression of intrinsic apoptosis molecule active (cleaved) caspase 9 (CASP9) by western blot analysis. FAS (0.7 fold), CASP8 (0.69 fold), and CASP3 (0.71 fold) expression was markedly down-regulated by MIR196B transfection (Fig. 3A). These results indicate that MIR196B regulates FAS-mediated apoptosis by directly down-regulating FAS in colorectal cancer cells. We further investigated the expression of FAS, CASP8, CASP3, and CASP9 by transfection of anti-MIR196B into SW480 cells (Fig. 3A). FAS and CASP3 expression did not change after anti-MIR196B transfection. This was expected because the level of endogenous MIR196B in SW480 cells was very low, as shown in Fig. 1C. However, anti-MIR196B transfection increased CASP8 (1.19 fold) expression slightly. We also investigated the expression of FasL (also called FASLG or CD95L) in MIR196B- or anti-MIR196B-transfected SW480 cells (Fig. 3A). FasL expression in SW480 cells was markedly down-regulated (0.7 fold) by MIR196B overexpression. However, there was no statistically significant change in FasL expression after anti-MIR196B transfection in SW480 cells. Interestingly, the FasL expression pattern was similar to the FAS expression pattern. These results suggest that MIR196B also regulates FasL expression, although we did not establish this in the present study.

### MIR196B directly regulates FAS expression

To further investigate the down-regulation of FAS by MIR196B, we assessed the expression of FAS and FAS-mediated active caspases after FAS monoclonal antibodie (mAb) treatment in SW480 cells. FAS, CASP8, and CASP3 expression in SW480 cells was up-regulated (1.21, 1.72 and 1.39 fold, respectively) by FAS mAb treatment (Fig. 3B). The up-regulation of these proteins was significantly attenuated by MIR196B (Fig. 3B). We also assessed the expression of FasL, NF-κB (p65), and ERK2 in FAS mAb-treated SW480 cells (Fig. 3C). Whereas FasL expression was up-regulated (1.23 fold) by FAS mAb treatment, NF-κB and ERK2 expression was slightly down-regulated (0.9 and 0.72 fold, respectively) by FAS mAb treatment (Fig. 3C). The up-regulation of FasL expression was attenuated by MIR196B transfection. Interestingly, NF-κB and ERK2 levels were up-regulated (1.54 and 1.41 fold, respectively) by MIR196B transfection (Fig. 3C).

### MIR196B down-regulates FAS-mediated molecules in HT29 cells

**Figure 4:**
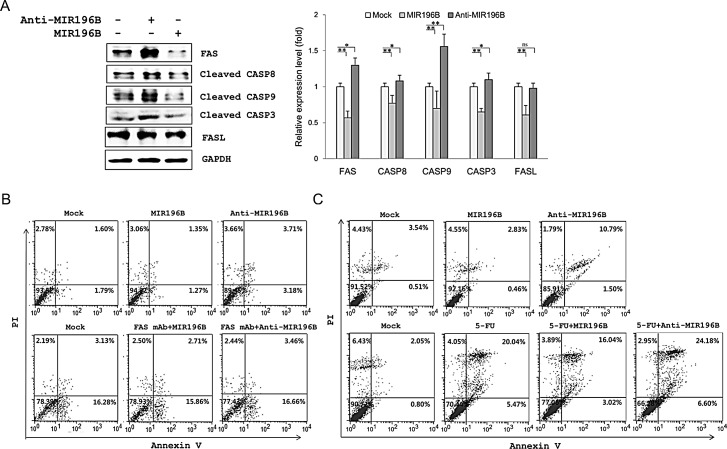
MIR196B regulates FAS-mediated apoptosis in SW480 or HT29 cells (A) Western blot analyses of FAS, FasL, and FAS-mediated molecules in MIR196B- or anti-MIR196B-transfected HT29 cells. Experiments were repeated four times and the *P* values were calculated by *t*-test (* *P* < 0.05; ** *P* < 0.01; ns = not significant). (B) Flow cytometry analysis of FAS-mediated apoptosis in MIR196B- or anti-MIR196B-transfected SW480 cells. Cells were cultured in the absence (upper panel) or presence (lower panel) of FAS mAb, stained with annexin V and PI, and analyzed by flow cytometry. The number in each box indicates the percentage of annexin V- and/or PI-positive cells. (C) Flow cytometry analysis of apoptosis in MIR196B- or anti-MIR196B-transfected HT29 cells. Cells were cultured in the absence (upper panel) or presence (lower panel) of 5-FU. Cells were collected 72 h after transfection, stained with annexin V and PI, and analyzed by flow cytometry. The number in each box indicates the percentage of annexin V- and/or PI-positive cells. Experiments were repeated three times with duplicates.

The endogenous MIR196B level in HT29 cells was higher than that in SW480 cells (Fig. 1C). Our results suggested that anti-MIR196B transfection would up-regulate FAS expression in HT29 cells. We investigated the expression of FAS and FAS-mediated caspases in MIR196B- or anti-MIR196B-transfected HT29 cells. As expected, the expression of FAS (1.3 fold), active CASP8 (1.08 fold), and active CASP3 (1.56 fold) was up-regulated by anti-MIR196B transfection (Fig. 4A). As observed in SW480 cells, FAS, CASP8, and CASP3 levels in HT29 cells were markedly down-regulated (0.57, 0.77 and 0.65 fold, respectively) by MIR196B transfection (Fig. 4A). Interestingly, the pattern of active CASP9 expression after MIR196B and anti-MIR196B transfection in HT29 cells differed from the pattern in SW480 cells. CASP9 expression levels were almost unchanged by MIR196B or anti-MIR196B transfection in SW480 cells (Fig. 3A), whereas CASP9 expression was remarkably up-regulated (1.56 fold) and down-regulated (0.7 fold) in HT29 cells by anti-MIR196B and MIR196B transfection, respectively (Fig. 4A). We also determined the expression of FasL in HT29 cells. FasL expression was markedly down-regulated (0.61 fold) by MIR196B in HT29 cells. However, whereas FAS expression was up-regulated by anti-MIR196B transfection in HT29 cells, FasL expression (0.98 fold) did not change (Fig. 4A).

### Effect of MIRI96B on Caspase 3/7 activities

We profiled and quantified, whether MIR196B can influence the activation of caspase 3/7 activities in SW480 and HT29 cells. As shown in Supplementary Figure 1D, MIR196B overexpression decreased caspase-3/7 activities in SW480 cells (0.80 fold change, p<0.01) and HT29 cells (0.89 fold change, p<0.05), compared to the mock condition, respectively.

### Effect of MIR196B or anti-MIR196B transfection on apoptosis in colorectal cancer cells

We analyzed apoptosis in MIR196B- or anti-MIR196B transfected colorectal cancer cell lines. As shown in Fig. 4B, apoptosis in SW480 cells increased (*P* < 0.05) after anti-MIR196B transfection. The increase (*P* < 0.01) in apoptosis was also observed in anti-MIR196B-transfected HT29 cells (Fig. 4C upper panel). The number of apoptotic cells among anti-MIR196B-transfected HT29 cells was much higher than among mock-treated cells. Although the ratio was low, the number of apoptotic cells decreased (*P* < 0.05) after MIR196B transfection in SW480 and HT29 cells (Fig. 4B and Fig. 4C). When FAS mAb was added to SW480 cells, apoptosis increased (Fig. 4B lower panel). The increased apoptotic cell ratio was not changed by MIR196B or anti-MIR196B transfection in FAS mAb-treated SW480 cells.

### Anti-MIR196B overexpression increased 5-FU-induced apoptosis in HT29 cells

We examined the IC_50_ values of 5-FU using MTT assay after the cells were exposed to 5-FU. The IC_50_ of 5-FU for HT29 was 4±0.02 μM/ml. We investigated whether anti-MIR196B or MIR196B played a functional role in 5-FU-induced apoptosis. The apoptosis rate was significantly increased (*P* < 0.01) in HT29 cells after 5-FU treatment compared to untreated control cells (Fig. 4C, lower panel). Anti-MIR196B or MIR196B overexpression increased (*P* < 0.01) or decreased (*P* < 0.05) the 5-FU-induced apoptosis in HT29 cells, compared to the cells treated with 5-FU, respectively. The result showed that the overexpression of anti-MIR196B up-regulated the 5-FU induced apoptosis in colon cancer cells (Fig. 4C, lower panel).

## DISCUSSION

miRNAs have been implicated as important regulators of gene expression in a variety of biological processes in various diseases, as well as in various diseases as tumor suppressor genes or oncogenes [9-11]. Using colon cancer tissues and normal colon tissues, we identified 31 colon cancer-associated miRNAs by miRNA microarray analysis and validated the results by qRT-PCR (Table 1 and Fig. 1A). We selected MIR196B for further study. MIR196B was up-regulated in human colorectal cancer tissue (Fig. 1B). Most functional studies of MIR196B have reported that the miRNA is deregulated in various leukemias [18-24]. We used mRNA microarray analysis and bioinformatics tools to identify MIR196B target genes in colorectal cancer.

FAS is a death domain-containing member of the TNF receptor superfamily [25]. FAS plays a central role in the physiological regulation of apoptosis, and it has been implicated in the pathogenesis of various malignancies [26]. In human cancer, tumor cells tend to downregulate FAS expression to avoid FAS-mediated apoptosis signaling [27, 28], particularly in metastatic human colorectal cancer [29, 30]. We showed that MIR196B regulates FAS and the FAS-mediated apoptosis pathway by directly downregulating FAS expression (Figs. 2,3, 4). These results suggest that MIR196B is a regulator of FAS-mediated apoptosis in colorectal cancer.

Apoptosis is a distinct cell death program that is primarily triggered by the intrinsic or extrinsic apoptosis pathway. The intrinsic pathway is activated by cytotoxic stress and is characterized by the release of cytochrome c from the mitochondria, resulting in the activation of downstream CASP9 and CASP3 [31]. The extrinsic pathway is activated by the binding of FasL to FAS, leading to CASP8 and CASP3 activation, which initiates multiple pro-apoptotic processes [32]. In this study, we showed that FAS, CASP8, and CASP3 expression in SW480 and HT29 cells was markedly down-regulated by MIR196B overexpression and that anti-MIR196B overexpression restored their expression (Fig. 3A and 4A). As we expected, the effect of anti-MIR196B was stronger in HT29 cells than in SW480 cells. The latter expressed lower levels of MIR196B than HT29 cells (Fig. 1C). Extrinsic pathway molecules were down-regulated by MIR196B in both cell lines, whereas the intrinsic pathway molecule CASP9 was regulated in a cell-type specific manner (Figs. 3A and B and 4A). Many miRNAs partially regulate target gene expression in specific cells or tissues. FAS was constitutively expressed at high levels in normal human colon tissues, but its expression was often reduced in human colorectal cancer tissues. Our results suggest that the reduction in FAS level was due to increased MIR196B expression in colorectal cancer. The reduction in FAS was not sufficient to induce apoptosis in colorectal cancer cells.

FAS is a critical component of the FAS-mediated apoptosis pathway and thus an attractive target in cancer therapy. FAS mAbs could act as effective anticancer agents by activating FAS-mediated apoptosis, as shown in the lower panel of Fig. 4B. However, FAS mAb induces extensive apoptosis in hepatocytes, resulting in lethal liver damage [33, 34], thus limiting the clinical use of FAS mAbs for systemic anticancer chemotherapy. As shown in Fig. 4B and Fig. 4C, MIR196B overexpression decreased the apoptotic cell ratio in SW480 and HT29 cells, whereas anti-MIR196B transfection increased apoptosis. These results indicate that MIR196B regulates the FAS-mediated apoptosis pathway by modulating FAS expression. Therefore, our results suggest that anti-MIR196B is a candidate material for anticancer therapy in human colorectal cancer. We also carried out the effect of MIR196B and anti-MIR196B with an anti-cancer agent 5-FU in HT29 cells (Fig. 4C, lower panel). These results indicated that anti-MIR196B increased the sensitivity of 5-FU, suggest that MIR196B may contribute to chemo-resistance in colon cancer.

FasL expression was only observed in colorectal carcinomas, but FAS was constitutively expressed at high levels in normal human colon tissues. However, FAS expression is often reduced in metastatic human colorectal carcinoma [29, 35]. FasL expression in tumor cells has been hypothesized as a “FAS counterattack,” by which tumor cells evade immune destruction by inducing FasL-mediated apoptosis in tumor-infiltrating lymphocytes [36]. However, this concept is legitimately debated [37, 38]. Interestingly, our results showed that FasL expression in SW480 and HT29 cells was markedly down-regulated by MIR196B overexpression (Fig. 3A and 4A). Furthermore, the up-regulation of FasL induced by FAS mAb treatment was attenuated by anti-MIR196B overexpression in SW480 cells (Fig. 3C). The FasL expression patterns in response to MIR196B and anti-MIR196B overexpression were similar to the FAS expression patterns (Fig. 3A and C and Fig. 4A). These results emphasizes that MIR196B regulates FasL expression by an unknown mechanism, although we did not establish this in the present study.

We measured NF-κB and ERK2 expression after MIR196B overexpression (Fig. 3C). Whereas FAS and FasL expression was down-regulated, NF-κB and ERK2 expression was up-regulated by MIR196B (Fig. 3C). Consequently, we assessed whether MIR196B regulated cell viability via the NF-κB or ERK2 pathway in SW480 and HT29 cells (Fig. S2). However, we did not find any evidence in association between MIR196B expression and cell proliferation in colorectal cancer cells.

In summary, we identified 31 miRNAs that were significantly up-regulated or down-regulated in colorectal cancer tissues. We investigated one of them, MIR196B, and identified 18 putative MIR196B target genes. *FAS* was verified as MIR196B targets in this study. MIR196B expression was up-regulated in colorectal cancer tissue, whereas FAS expression was down-regulated. MIR196B directly repressed FAS expression in colorectal cells. In contrast, anti-MIR196B up-regulated FAS expression and increased apoptosis in colorectal cancer cell lines. Our results suggest that up-regulated MIR196B modulates apoptosis in colorectal cancer cells by partially repressing FAS expression and that anti-MIR196B could be useful as an anti-cancer drug in colorectal cancer.

## MATERIALS AND METHODS

### Patients and tissue samples

The tissue samples used in this study were provided by the Biobank of Wonkwang University Hospital, a member of the National Biobank of Korea. With approval from the institutional review board and informed consent, we obtained colon cancer tissue from 14 colon cancer patients (7 males and 7 females) and rectal cancer tissue from 2 rectal cancer patients (2 males). The mean ages of the colon cancer patients and rectal cancer patients were 65.6 years and 72 years, respectively. Four colon cancer tissue samples and matched normal colon tissue samples (2 males and 2 females) were used for miRNA microarray analysis. Three separate colon cancer tissue samples and matched normal colon tissue samples (1 male and 2 female) were used to validate the miRNA microarray results. In addition, one separate colon cancer tissue sample with matched normal colon tissue sample and two rectal cancer tissue samples with matched normal rectal tissue samples were used to assess MIR196B expression. Six separate colon cancer tissue samples and matched normal colon tissue samples (4 males and 2 females) were used to analyze FAS protein expression.

### Cell culture

The human colorectal cancer cell lines SW480, HT29, and Caco2 were obtained from Korea Cell Line Bank. SW480 and HT29 cells were cultured in RPMI 1640 (HyClone, Logan, UT, USA) supplemented with 10% FBS in 5% CO_2_ at 37°C in a humidified atmosphere. Caco2 cells were cultured in Alpha-MEM (HyClone) supplemented with 20% FBS in 5% CO_2_ at 37°C in a humidified atmosphere.

### miRNA expression profiling

Total RNA (100 ng) was hybridized to an Agilent Human microRNA Microarray (Agilent Technologies, Santa Clara, CA, USA). MicroRNA was labeled, hybridized, and washed following Agilent's instructions. Images of hybridized microarrays were acquired with a DNA microarray scanner (Agilent Technologies), and the microarray images were analyzed with Feature Extraction software (Agilent Technologies). The standard of statistical significance was the corrected ratio of the hybridization signal intensity in colon tumor tissue to the hybridization signal intensity in normal tissue.

### RNA extraction and quantitative RT-PCR

Total RNA was isolated with TRIzol reagent (Invitrogen, Carlsbad, CA, USA) according to the manufacturer's protocol. After digestion with DNase and cleanup, RNA samples were quantified, aliquoted, and stored at −80°C. Total RNA isolated from tissue samples and/or cultured cells were used as a template to synthesize cDNA for quantitative RT-PCR (qRT-PCR) analysis in a StepOne Real-time PCR system (Applied Biosystems, Foster City, CA, USA).

The differential miRNA expression patterns were validated with the TaqMan qRT-PCR assay (Applied Biosystems) or the NCode VILO miRNA cDNA Synthesis kit for qRT-PCR and EXPRESS SYBR GreenER miRNA qRT-PCR kit (Invitrogen). qRT-PCR with SYBR Green dye (Applied Biosystems) was used to assess mRNA expression. RNU48 (for TaqMan qRT-PCR) or 5.8S (for SYBR qRT-PCR) and *GAPDH* were used as endogenous controls for qRT-PCR of miRNA and mRNA, respectively. Each sample was run in triplicate.

### MIR196B transfection

SW480 and HT29 cells (3 × 10^5^) or Caco2 cells (1.5 × 10^5^) were plated in 6-well or 10-cm culture plates and cultured as described above. MIR196B (hsa-miR-196b, Pre-miR miRNA Precursor AM17100, Product ID: PM12946) or anti-MIR196B (anti-hsa-miR-196b, Anti-miR miRNA Inhibitor AM17000, Product ID: AM12946) were commercially synthesized (Ambion, Austin, TX, USA) and transfected at 50 nmol/L using Lipofectamine RNAiMAX (Invitrogen) or siPORT™ *NeoFX*™ transfection agent (Ambion) according to the manufacturers' recommendations. Cells were harvested 24–48 h (for miRNA and mRNA) or 48–72 h (for protein) after transfection for functional assays, flow cytometry assays, or RNA/protein extraction.

### mRNA expression profiling of MIR196B target genes

SW480 and Caco2 cells were transfected with MIR196B or MIR1 as a control. Total RNA was isolated 48 h after transfection, amplified, and purified using the Illumina TotalPrep RNA Amplification Kit (Ambion) according to the manufacturer's instructions to yield biotinylated complementary RNA (cRNA). Labeled cRNA samples (750 ng) were hybridized to a HumanHT-12 v4 Expression BeadChip array (Illumina, Inc., San Diego, CA, USA) for 16–18 h at 58°C. The array signal was detected using Amersham fluorolink streptavidin-Cy3 (GE Healthcare Bio-Sciences, Little Chalfont, UK), following the instructions in the BeadChip array manual. Arrays were scanned with an Illumina BeadArray Reader according to the manufacturer's instructions. Array data was exported, processed, and analyzed using Illumina BeadStudio v3.1.3 (Gene Expression Module v3.3.8). Array data were filtered for a detection p-value < 0.05 (similar to signal-to-noise) in at least 50% samples.

### MIR196B target prediction by bioinformatics methods

The miRNA targets were predicted using the computer-aided algorithms TargetScan (http://www.targetscan.org) and miRWalk (http://www.umm.uni-heidelberg.de/apps/zmf/mirwalk/index.html).

### Plasmid constructions and luciferase assays

Wild-type or mutant fragments of the 3′ untranslated region (UTR) of *FAS* containing the predicted binding site for MIR196B, were amplified by PCR using the primer set shown in Table S2. The PCR product was cloned into the pmirGLO Dual-Luciferase miRNA Target Expression Vector (Promega, Madison, WI, USA).

For reporter assays, cells (5 × 10^4^/well) were seeded in 24-well plates and co-transfected with wild-type or mutant FAS constructs (500 ng/well) or with 50 nM MIR196B or MIR-1 (negative control) using Lipofectamine 2000 (Invitrogen Life Technologies) and siPORT™ *NeoFX*™ Transfection Agent (Ambion), respectively, according to manufacturers' instructions. Firefly and *Renilla* luminescence was measured 24 h after transfection using the Dual-Glo Luciferase Assay System (Promega) according to the manufacturer's instructions. Non-transfected cells were used for background subtraction, and the ratio of firefly reporter luminescence to *Renilla* reporter luminescence (control) was calculated. All experiments were performed in triplicate and repeated at least three times.

### Antibodies and western blot analysis

SW480 or HT29 cells (2 × 10^5^ cells/well) were seeded in 6-well plates and incubated for 72 h. Whole cell lysates were prepared by incubation in RIPA buffer supplemented with a protease inhibitor mixture for 30 min at 4°C. Protein was collected by centrifugation at 12,000 rpm for 30 min at 4°C. Equal amounts of protein (50 μg; determined by the Bradford assay) were boiled in Laemmli buffer, subjected to 12.0% or 15.0% SDS-PAGE, and transferred to PVDF membranes. The membranes were incubated with blocking buffer [5% BSA in TBS containing 0.1% Tween-20 (TBS-T)] for 2 h at room temperature. Membranes were then incubated overnight at 4°C with primary antibodies to FAS (G-9), FasL (C-178), ERK1/2 (H-72), NF-κB p65 (Santa Cruz Biotechnology), caspase-9 (human specific; #9502), caspase-8 (1C12; #9746) (Cell Signaling Technology, Boston, MA, USA) and caspase-3 (Enzo Life Sciences) and washed three times for 10 min per wash with TBS-T. Membranes were incubated with HRP-conjugated rabbit or mouse IgG secondary antibodies for 1 h at room temperature. After washing three times for 10 min in TBS-T, protein was detected with ECL solution (Millipore Corporation, Billerica, MA, USA) and a FluorChem E System (Cell Biosciences, Santa Clara, CA, USA). After protein detection, some membranes were stripped with stripping buffer for 1 h at room temperature and re-probed with antibody to GAPDH (0411; Santa Cruz Biotechnology), used as a loading control. Protein expression was evaluated using ImageJ software (version 1.44; http://rsbweb.nih.gov/ij/index.html).

### Caspase 3/7 assay

Caspase 3 and 7 activation assays were performed using the Caspase-Glo 3/7 Assay Kit (Promega) according to the manufacturer's instructions. Briefly, the cells were seeded in 96-well plates at a density of 2 × 10^4^ cells/well and transfected with MIR196B or control. After 48h of transfection, Caspase-Glo 3/7 Reagent (100μl) was added to each well and mixed gently using a plate shaker at 500 rpm for 30 sec. The plate was then incubated at room temperature for 2 h in the dark. The luminescence of each sample was measured by using a GloMax®-Multi+ Detection System (Promega). The data were analyzed with GraphPad Prism 5 (GraphPad Software Inc., San Diego, USA).

### Viability (MTT) assay

The effect of MIR196B and antisense MIR196B (anti-MIR196B) on cell viability was evaluated with the MTT (3-(4,5-dimethylthiazol-2-yl)-2-5-diphenyltetrazoliumbromide) assay (M2128; Sigma-Aldrich, St. Louis, MO, USA). SW480 or HT-29 cells (2 × 10^4^ cells/ well) were transfected with 50 nM MIR196B or anti-MIR196B in a 96-well plate and grown for 72 h. The medium was carefully removed, and cells were washed twice with 1× PBS. MTT solution (5 mg/ml), dissolved in culture medium at a final concentration of 0.5 mg/ml, was added to each well. The plates were wrapped in aluminum foil and incubated for another 3 h at 37°C. The medium was removed, and 100 μl of DMSO (Duchefa Biochemie, Haarlem, The Netherlands) was added to solubilize the MTT tetrazolium crystals. Finally, the solution was agitated with a pipette until no change in color was observed, and the optical density was read at 560 nm (OD560) using a GloMax®-Multi+ Detection System (Promega). The percentage of viable cells was estimated by comparison with the untreated controls. At least three independent experiments were performed. The IC_50_ (50% inhibitory concentration) values of 5-FU (F6627, Sigma-Aldrich Co., Missouri, USA) was assessed by MTT assay. The molar concentration required for 50% inhibition of cell viability (IC_50_) was calculated. In the following experiments, the concentration of 5-FU used were equal to the IC_50_ and duration of 5-FU treatment was 72h in HT29 cells.

### Apoptosis analysis (flow cytometry)

Apoptosis was analyzed by flow cytometry with the Annexin V-FITC Apoptosis Detection Kit according to the manufacturer's instructions (Sigma-Aldrich). Briefly, SW480 or HT29 cells (1 × 10^5^/well or 3 × 10^5^/well) were seeded in 6-well plates and collected 72 h after transfection with MIR196B or anti-MIR196B or control. SW480 cells (3 × 10^5^/well) were also seeded in 6-well plates, cultured 24 h with FAS mAb (anti-FAS/CD95 monoclonal antibody; MBL, Nagoya, Japan), and collected 48 h after transfection with MIR196B, anti-MIR196B, or control. 5-FU (4μM/ml) was added 10h after transfection and the cells were incubated for 72 h. The cells were trypsinized, washed in cold PBS, and re-suspended in 100 μl 1× binding buffer. To estimate the apoptotic cell number, cells were double-stained with annexin V and propidium iodide (PI) solution, incubated for 20 min on ice, and resuspended in 1× PBS (300 μl). Samples were then analyzed using a FACSCalibur (Becton Dickinson, USA) and CellQuest analysis software (Becton Dickinson). For each sample, 10,000 cells were analyzed.

### Statistical analysis

Each experiment was repeated at least three times with consistent results. All of the data were represented as mean ± standard deviation (SD). Statistical differences were analyzed by GraphPad prism 5.0 statistical software (GraphPad Software Inc., San Diego, USA) or Student's t-test, and p-values of less than 0.05 (p<0.05) were regarded as statistically significant.

## SUPPLEMENTARY MATERIAL FIGURES AND TABLES


