# Supplementary material for: MicroRNA 196B regulates FAS-mediated apoptosis in colorectal cancer cells
Source: Oncotarget. 2014 Dec 26;6(5):2843–55. doi: 10.18632/oncotarget.3066 (PMC4413621; doi:10.18632/oncotarget.3066)
Supplement: Supplementary file 1 [file oncotarget-06-2843-s001.pdf]

MicroRNA 196B regulates FAS-mediated apoptosis in colorectal cancer cells

Supplementary Material

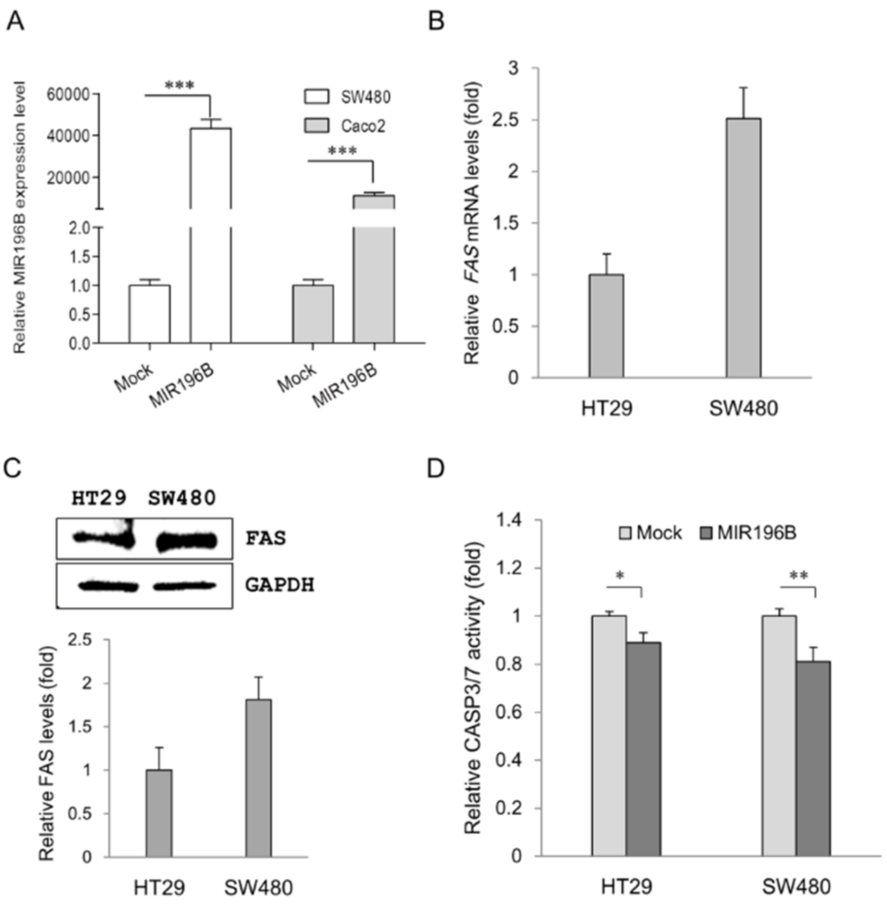

Figure S1: MIR196B transfection efficiency, FAS levels in colon cancer cell lines, and CASP3/7 levels by MIR196B

(A) SW480 or Caco2 cells were transfected with MIR196B. Cells were collected at 24 h to confirm the transfection efficiency. Data assessed from three independent experiments and the *P* values were calculated by *t*-test (*** $P < 0.001$). (B) qRT-PCR analysis and (C) western blot analysis in SW480 and HT29 cells. (D) MIR196B regulated caspase 3/7 activities in SW480 and HT29 cells. At 48 h after transfection of MIR196B, there were significantly decreased in caspase 3/7 activities compared to mock conditioned cells, respectively. The *P* values were calculated by *t*-test (* $P < 0.05$; ** $P < 0.01$).

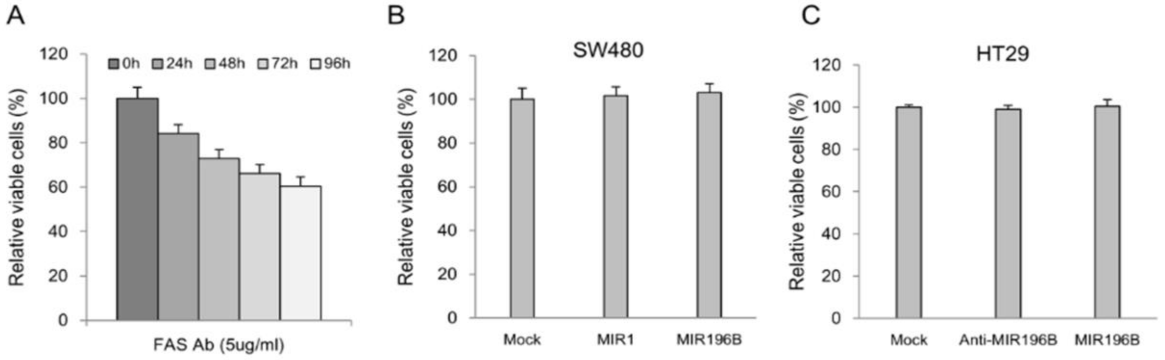

Figure S2: The effect of MIR196B on cell proliferation in human colon cancer cells

(A) SW480 cells (2×10^4 cells/ well) were cultured with FAS mAb (5 μg/ml) for 96 h. Cells were collected at 24 h intervals for the MTT assay. SW480 cells (B) or HT-29 cells (C) were transfected with 50 nM MIR196B, anti-MIR196B, or MIR1 in a 48-well plate and grown for 72 h. The relative proliferation of cells transfected with each miRNA precursor was compared with the proliferation of mock-transfected control cells.

Table S1: MIR196B candidate target genes identified by mRNA microarray analysis

Gene symbol	Accession	Gene name	Chromosome location
AIF1L	NM_031426.2	allograft inflammatory factor 1-like	9q34.13
ANKDD1A	NM_182703.3	ankyrin repeat and death domain containing 1A	15q22.31
ANXA1	NM_000700.1	annexin A1	9q21.13
APEX2	NM_014481.2	APEX nuclease 2	Xp11.21
ATP6V0B	NM_004047.3	ATPase, H+ transporting, lysosomal 21kDa, V0 subunit b	1p32.3
B3GALT6	NM_080605.3	UDP-Gal:betaGal beta 1,3-galactosyltransferase polypeptide 6	1p36.33
BTG3	NM_006806.3	BTG family, member 3	21q21.1
C14orf109	NM_015676.1	transmembrane protein 251	14q32.12
C7orf23	NM_024315.2	transmembrane protein 243, mitochondrial	7q21.12
C7orf55	NM_197964.3	chromosome 7 open reading frame 55	7q34
C7orf59	NM_001008395.2	late endosomal/lysosomal adaptor, MAPK and MTOR activator 4	7q22.1
CCDC72	NM_015933.3	translation machinery associated 7 homolog	3p21.31
CCND3	NM_001760.2	cyclin D3	6p21
CTSZ	NM_001336.2	cathepsin Z	20q13.32
DDX26B	NM_182540.3	DEAD/H (Asp-Glu-Ala-Asp/His) box polypeptide 26B	Xq26.3
DIRAS2	NM_017594.3	DIRAS family, GTP-binding RAS-like 2	9q22.2
DLX1	NM_001038493.1	distal-less homeobox 1	2q32
EEF1A1	NM_001402.5	eukaryotic translation elongation factor 1 alpha 1	6q14.1
FAS	NM_152877.1	cell surface death receptor	10q24.1
GLRX	NM_002064.1	glutaredoxin (thioltransferase)	5q14
GLTP	NM_016433.3	glycolipid transfer protein	12q24.11
H3F3B	NM_005324.3	H3 histone, family 3B	17q25.1

HADH	NM_005327.2	hydroxyacyl-CoA dehydrogenase	4q22-q26
HAND1	NM_004821.1	heart and neural crest derivatives expressed 1	5q33
HBXIP	NM_006402.2	late endosomal adaptor, MAPK and MTOR activator 5	1p13.3
HNRPDL	NR_003249.1	heterogeneous nuclear ribonucleoprotein D-like	4q21.22
HOXA5	NM_019102.2	homeobox A5	7p15.2
HOXA9	NM_152739.3	homeobox A9	7p15.2
HOXB6	NM_018952.4	homeobox B6	17q21.3
HOXB7	NM_004502.3	homeobox B7	17q21.3
HOXC8	NM_022658.3	homeobox C8	12q13.3
HSD17B10	NM_004493.2	hydroxysteroid (17-beta) dehydrogenase 10	Xp11.2
IFI6	NM_022873.2	interferon, alpha-inducible protein 6	1p35
KCNIP3	NM_013434.4	Kv channel interacting protein 3, calsenilin	2q21.1
LAGE3	NM_006014.3	L antigen family, member 3	Xq28
LOC100132805	XM_001719188.1	Homo sapiens similar to predicted protein	chr. 3
LOC400455	XR_018793.1	Homo sapiens hypothetical LOC400455	chr. 15
LOC645058	XM_930423.1	similar to hepatitis B virus x-interacting protein (LOC645058)	Chr. 1
LRRC49	NM_017691.2	leucine rich repeat containing 49	15q23
MED10	NM_032286.2	mediator complex subunit 10	5p15.31
MRPL39	NM_017446.3	mitochondrial ribosomal protein L39	21q21.3
MYBBP1A	NM_014520.2	MYB binding protein (P160) 1a	17p13.3
NHP2	NM_001034833.1	NHP2 ribonucleoprotein	5q35.3
NOTCH1	NM_017617.3	notch 1	9q34.3
NUDT22	NM_032344.1	nudix (nucleoside diphosphate linked moiety X)-type motif 22	11q13.1
OSBPL8	NM_001003712.1	oxysterol binding protein-like 8	12q14
PABPC4	NM_003819.2	poly(A) binding protein, cytoplasmic 4	1p34.2
PDE6D	NM_002601.2	phosphodiesterase 6D, cGMP-specific, rod, delta	2q35-q36
PHLDA2	NM_003311.3	pleckstrin homology-like domain, family A, member 2	11p15.4
PPP1CB	NM_206876.1	protein phosphatase 1, catalytic subunit, beta isozyme	2p23
PRKAG2	NM_024429.1	protein kinase, AMP-activated, gamma 2 non-catalytic subunit	7q36.1
RASL11B	NM_023940.2	RAS-like, family 11, member B	4q12
RBP1	NM_002899.2	retinol binding protein 1, cellular	3q23
RNASEH2A	NM_006397.2	ribonuclease H2, subunit A	19p13.2
S100A3	NM_002960.1	S100 calcium binding protein A3	1q21
S100A4	NM_019554.2	S100 calcium binding protein A4	1q21
SCNN1A	NM_001038.4	sodium channel, non-voltage-gated 1 alpha subunit	12p13
SERBP1	NM_001018069.1	SERPINE1 mRNA binding protein 1	1p31
SPRED1	NM_152594.1	sprouty-related, EVH1 domain containing 1	15q14
TGFB1I1	NM_015927.3	transforming growth factor beta 1 induced transcript 1	16p11.2
TMEM2	NM_013390.1	transmembrane protein 2	9q21.13
TNNC1	NM_003280.1	troponin C type 1	3p21.1
TPD52	NM_005079.2	tumor protein D52	8q21.13
TSPO	NM_000714.4	translocator protein (18kDa)	22q13.31
UFSP1	NM_001015072.3	UFM1-specific peptidase 1	7q22.1
VSNL1	NM_003385.4	visinin-like 1	2p24.3
ZNF512	NM_032434.2	zinc finger protein 512	2p23

Table S2: Primer sequences used for qRT-PCR analysis and luciferase assays in this study

Applications	Primers	Primer sequence (5' → 3')
qRT-PCR	FAS-QF1	ATGGCCAATTCTGCCATAAG
	FAS-QR1	GACAAAGCCACCCCAAGTTA
Luciferase assay	FAS-LF1	CAGCTCGAGCTACCTCAAAGACCTTTGCAC
	FAS-LR1	CAGCTCGAGTATTTCCATGTTTGGGGGTGC
	FAS-LF2	CTGAGAAAGAAGTGAGCCAGTAAAA
	FAS-LR2	TTTTACTGGCTCACTTCTTTCTCAG
